# Eco-Friendly, Multi-Mode Processable Highly Moldable Wood Enabled by the Reconstruction of Hydrogen-Bonding Domain

**DOI:** 10.1007/s40820-026-02121-y

**Published:** 2026-03-04

**Authors:** Rui Yang, Linghui Qi, Xiaoli Wu, Zhipeng Liu, Huiyang Bian, Changlei Xia, Changtong Mei, Shuaicheng Jiang, Meng Yao, Jianzhang Li

**Affiliations:** 1 Jiangsu Co-Innovation Center of Efficient Processing and Utilization of Forest Resources, International Innovation Center for Forest Chemicals and Materials, College of Materials Science and Engineering, Nanjing Forestry University, Nanjing, 210037 People’s Republic of China; 2 School of Mechanical and Electronic Engineering, Nanjing Forestry University, Nanjing, 210037 People’s Republic of China; 3 Jiangsu Provincial Key Lab of Sustainable Pulp and Paper Technology and Biomass Materials, Nanjing Forestry University, Nanjing, 210037 People’s Republic of China; 4 State Key Laboratory of Efficient Production of Forest Resources & MOE Key Laboratory of Wood Material Science and Application, Beijing Forestry University, Beijing, 100091 People’s Republic of China; 5 College of Materials Science and Engineering, Sichuan University, Chengdu, 610065 People’s Republic of China

**Keywords:** Wood modification, 3D structures, Flexible and moldable wood, Reconstruction of hydrogen-bonding domains

## Abstract

The production of advanced 3D engineering materials relies on energy-intensive moldable materials such as metals and plastics, making it difficult to cope with the increasingly severe global energy crisis. Wood, as a sustainable material, can be molded through hydrothermal treatment, but the limited plasticity hinders its ability to manufacture precision devices. Herein, the process of hydrogen-bond domain reorganization is used in the manufacture of highly moldable wood to enhance the plasticity of wood and ensure the stability of the cellulose structure. The native hydrogen-bond network in the wood cell wall is disrupted and liberated the cellulose fibril matrix through delignification. Subsequent epoxidized soybean oil acrylate (AESO) plasticization enables significantly enhanced plasticity. Hydrogen-bond domains between fibers are reconstructed through moisture variation. Meanwhile, AESO forms a protective layer on the surface of the fibers, preventing excessive moisture from entering and causing the collapse of the fiber framework. This process allows the material to be shaped into complex 3D geometries, including origami cranes or honeycombs, through low-energy hydrothermal processing. This strategy addresses both dimensional stability challenges and environmental instability associated with wood composite materials and offers an eco-friendly alternative to functionalized structures in aviation and transportation.

**Supplementary Information:**

The online version contains supplementary material available at 10.1007/s40820-026-02121-y.

## Highlights

- A hydrogen-bond reconstruction strategy creates a moldable wood with greatly improved dimensional stability (≈ 80% lower moisture absorption) and plasticity for precision 3D shaping.
- We develop a moldable wood that combines high plasticity with exceptional water resistance, enabling the creation of complex, precise 3D structures like mechanical metamaterials.
- This moldable wood bridges sustainable materials and precision engineering, offering an energy-efficient alternative to synthetic composites in demanding fields like aviation.

**Supplementary Information:**

The online version contains supplementary material available at 10.1007/s40820-026-02121-y.

## Graphical abstract

**Figure:**
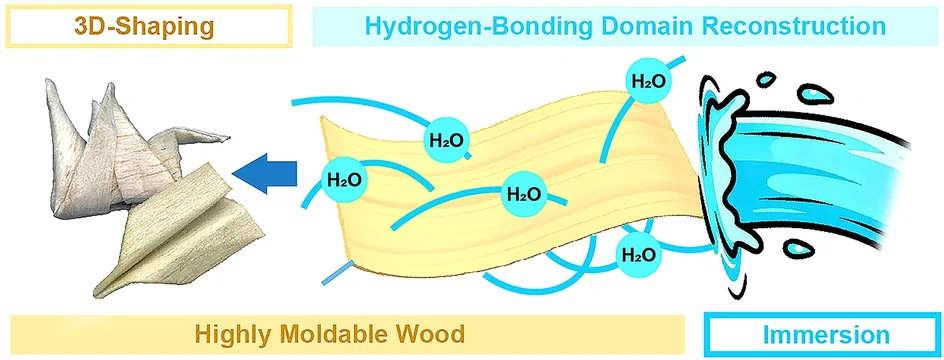

## Introduction

With the accelerating pace of industrial modernization, 3-dimensional (3D) engineering structures across aerospace [1, 2], civil infrastructure [2–4], and biomedical devices [5, 6] are increasingly demanding structural materials that integrate complex architectures and multifunctional properties. Typical approaches encompass morphological engineering utilizing organic polymers (e.g., thermoplastics, aramid fibers) and metallic systems (e.g., gold, silver, alloys) via extrusion, injection molding, textile processing, origami technology and 3D-printing. Nevertheless, these conventional manufacturing routes are frequently plagued by substantial energy consumption, multi-step processing complexities, and environmental concern, which critically hinder their applicability in the context of sustainable development [7–9].

Wood, as the natural composite with hierarchical architecture, exhibits unique moldability mechanisms [10–12]. Unlike metals or plastics requiring high-energy bond cleavage (metallic bonds: 100–800 kJ mol^−1^; covalent bonds: 200–400 kJ mol^−1^), the hierarchical structure of wood relies on reversible hydrogen-bonding networks [13] (5–30 kJ mol^−1^) between cellulose fibrils and lignin–hemicellulose matrices [14]. This enables plasticity molding through low energy hydrothermal treatment rather than energy-intensive melting. Additionally, wood is an environmentally friendly material, unlike plastics that can cause long-term pollution and metals whose extraction and processing often have a heavy environmental footprint [15, 16].

In previous studies [17–19], moldable wood was produced through delignification and hydrothermal treatment. These flexible wood chips were typically used for larger structures due to the inherent hygroscopicity and anisotropy of wood. Combined with persistent high-strength hydrogen-bond domains in crystalline cellulose regions, flexible wood chips were not suitable for precise manufacturing. Under extreme conditions such as aerospace applications or long-haul transportation, materials may face critical challenges from significant thermal gradients and humidity differentials [20]. These environmental extremes induce pronounced dimensional instability in conventional moldable wood, ultimately resulting in product failure. Moreover, although moisture-induced plasticity is inherent to wood and has been exploited in prior moldable wood systems, precise control over the softening process still remains challenging. Excessive water leads to structural failure, whereas insufficient moisture limits shapability. Thus, although wood demonstrates exceptional sustainability, there are still certain limitations in the manufacturing of advanced 3D structures for engineering applications that require urgent resolution.

**Fig. 1:**
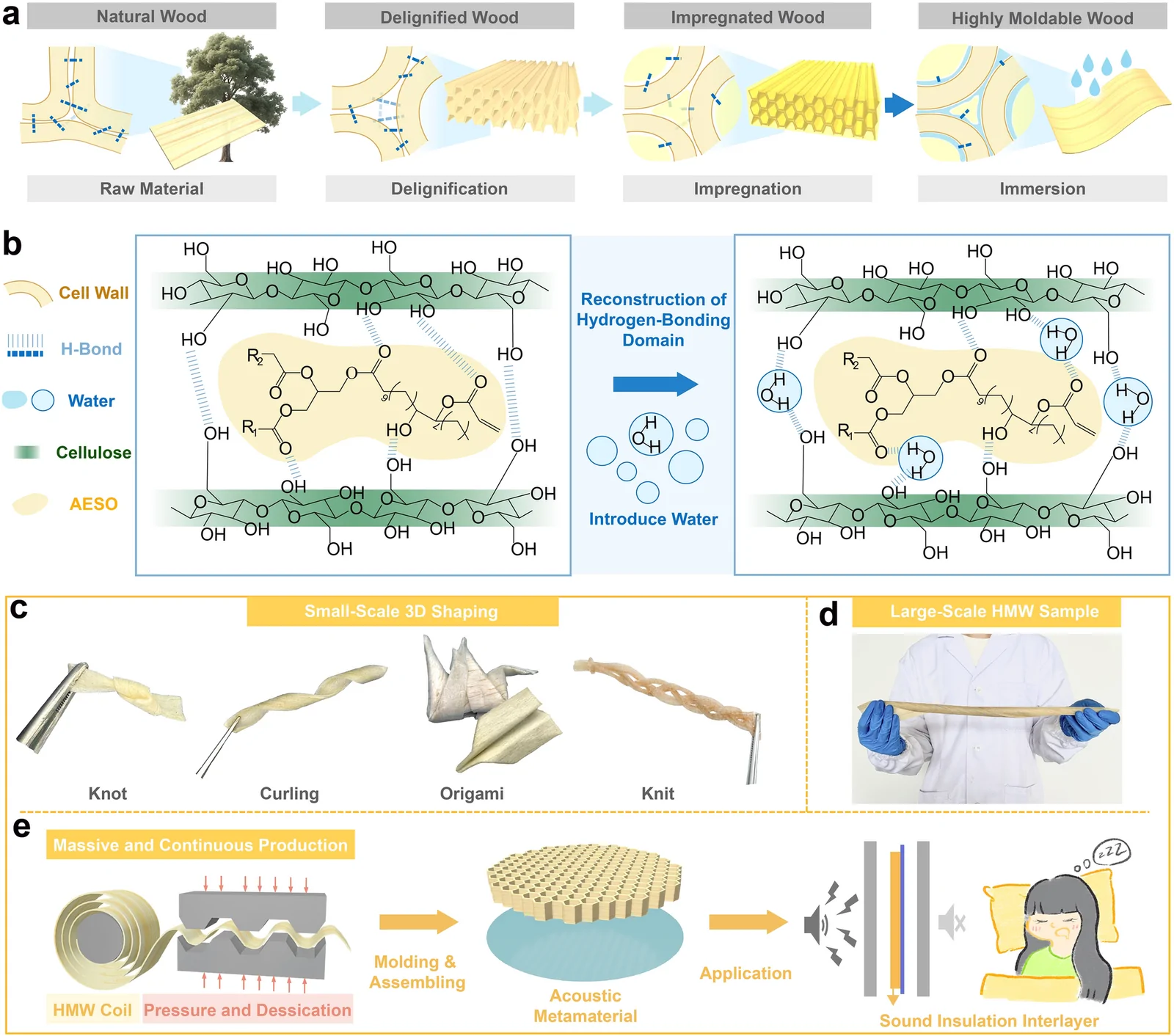
Preparation steps and applications of HMW. **a** Delignification, AESO impregnation and water immersion process. **b** Chemical composition and hydrogen-bond network reconstruction of HMW. **c** Small-scale 3D shaping examples of HMW. **d** Large-scale HMW sample. **e** Massive and continuous production strategy and the application of acoustic metamaterial made by HMW

Herein, we present a hydrogen-bond (H-bond) reconstruction strategy to fabricate highly moldable wood (HMW) with greatly improved dimensional stability and plasticity. This strategy moves beyond simple water-assisted molding by introducing hydrogen-bond domain reconstruction mediated by a bio-based resin. Through delignification and epoxidized soybean oil acrylate (AESO) impregnation (Fig. 1a), we construct a covalently crosslinked matrix that significantly reduces moisture absorption by approximately 80% while enabling precise shaping (Movie S1). AESO forms stronger hydrogen bond with cellulose, recreating a hydrogen-bonding network while preserving plasticity (Fig. 1b). Impregnating delignified wood with (AESO) can expand the wet-processing window by modulating water-fiber interactions. The hydrophobic segments of AESO reduce the hygroscopic sensitivity of cellulose, preventing over-softening, while during drying, AESO facilitates the reconstruction of a stabilized, dense hydrogen-bond network between fibers. This property of HMW makes it possible to achieve high-precision 3D shaping (Fig. 1c), such as knotting, curling, origami and knitting, and large-scale manufacturing (Fig. 1d). We used HMW to prepare some special structures, which demonstrated the unique effects of mechanical metamaterials (Fig. 1e) [21, 22]. This breakthrough addresses the shortage of wood in the manufacture of precision devices, bridges the gap between sustainable materials and precision engineering, offering an energy-efficient alternative to synthetic honeycombs in aviation and transportation.

## Experimental Section

### Materials

Balsa wood (Ochroma lagopus Swartz) was purchased from Yudong Composite Materials Co., Ltd. in Suzhou, China. Sodium hydroxide, sodium sulfite, and distilled water were purchased from Nanjing Chemical Reagent Co., Ltd. Epoxy soybean propylene glycol resin was purchased from Youming (Shanghai Chemical Co., Ltd).

### Preparation of Delignified Wood (DW), HMW and Honeycomb Thin-Film Acoustic Structures

#### Preparations of the DW

The balsa wood samples were used after they were dried at 103 °C for 4 h. At room temperature, a mixture of sodium hydroxide and sodium sulfite (molar ratio of 2.5:0.4) was added to water and placed in an oil bath until the solid particles dissolved, forming a uniform, transparent solution. The balsa samples were immersed in the mixture for 9 h. The wood sample was removed from the mixture and washed with water for 12 h. The pH of the washing water was measured with a pH paper, which showed neutrality. Then, the sample was frozen for 48 h and placed in a vacuum freeze dryer for drying treatment until the wood chips are completely dry.

#### Preparations of the HMW

Epoxidized soybean oil acrylate (AESO) was dissolved in acetone solution and stirred until the solid was completely dissolved to prepare 10%, 20%, 30%, and 40% AESO diluted solutions with respective mass fractions. Parallel and non-overlapping pretreated wood samples were immersed in the diluted AESO solution and subjected to vacuum at a negative pressure of -0.1 MPa for 24 h to produce impregnated wood. After vacuum impregnation, the wood samples were removed and soaked in deionized water at 25 °C without stirring for 10 min to obtain f-HMW. In this work, we refer to the material produced by our strategy as highly moldable wood (HMW), to distinguish it from conventional moldable wood (MW) systems that rely primarily on delignification.

#### Preparations of the Honeycomb Thin-Film Acoustic Structures

The prepared f-HMW was placed in a mold and shaped by drying to form a wood-based functional material. It was assembled into a honeycomb-shaped thin film acoustic material structure. The honeycomb structure and the film were bonded into a single unit using a 2% mass ratio of photo-curable adhesive polyethylene glycol diacrylate. Then, the bonded unit was cured in a UV curing chamber for 30 s, and the resulting structure, with a thickness of 2 mm and a diameter of 100 mm, forms a honeycomb-shaped thin film acoustic material.

### Characterization

L-NMR test was conducted using VTMR20-010-T, proton spin relaxation time (T_2_) measurements of the hydrogel were conducted at a proton resonance frequency of 21 MHz (0.5 T). The ATR spectrum of the sample was evaluated in the range of 4000–500 cm^−1^ using a Fourier transform infrared (FTIR) spectrometer (Nicolet iS50, Thermo Fisher Scientific, USA). The scanning frequency was 10 kHz, and the scanning time was 16 s.

The X-ray diffraction (XRD) test revealed the crystalline structures before and after the delignification and superhydrophobic coating. The samples were scanned by the Discovery Diffractometer (DSC250, TA Instruments, USA) from 5° to 40° (2θ). The crystallinity index (CrI) of cellulose was calculated from the XRD patterns using the empirical Segal method. The intensity of the (002) diffraction peak at approximately 22.5° and the minimum intensity attributed to the amorphous region at approximately 18° were used in the following equation:

1 $${\mathrm{CrI}}\left( \% \right) = \left[ {\left( {I_{002} - I_{{{\mathrm{am}}}} } \right)/I_{002} } \right] \times 100\%$$

*I*₀₀₂: Represents the maximum diffraction intensity of the crystal plane of cellulose *I* crystal form (002). This peak is located near 2θ ≈ 22.5°.

*I*am: Representing the scattering intensity in the amorphous region, the minimum intensity value near 2θ ≈ 18° is usually taken.

Small-angle X-ray scattering measurements were conducted using a NanoSTAR small-angle X-ray scattering (SAXS) system (Bruker AXS). The tensile strength of the samples was tested using an AGS-X Shimadzu (Japan) universal testing machine, and each sample was tested three times. The sound insulation performance of the sample was tested using an impedance tube. The average value is taken after each sample is tested three times. Regular-shaped and smaller-sized samples were selected for testing, and the sound transmission loss (STL) was measured using the four-microphone method. The dimensional stability test was conducted following the GB/T 1932–2009 standard.

The WCA of each sample was measured three times to obtain an average value. The test was conducted using an instrument manufactured by KRUSS of Germany. For each sample, three different points were selected for testing, and the average value was taken.

#### COMSOL Simulation

A three-dimensional geometric model of the sample is first created, with dimensions identical to the tested samples. Fluid domains are established both in front of and behind the sample model, as well as within the honeycomb cavities, and the entire geometry forms a unified assembly. Physical fields are assigned to distinct regions of the geometric model. The fluid domains are configured as pressure acoustic fields, while the frame and membrane of the sample are defined as solid mechanics fields. The interfaces between the fluid domains and the sample are designated as acoustic-structure boundaries. The degrees of freedom at the sample boundaries are constrained using the prescribed displacement boundary. The fluid domain boundaries are set to hard acoustic boundaries by default. To enhance computational efficiency, the effects of random sound wave incidence are neglected. The fluid domains are configured with plane wave radiation, where the incident sound pressure is set to 1 Pa and directed perpendicular to the sample. Material parameters are assigned based on experimental data. The fluid domains are defined as air, while the solid mechanics fields adopt material properties corresponding to the actual sample. Key material properties used in this study are listed in Table 1. To improve computational accuracy, appropriate damping (isotropic loss factor) is applied to the membrane and solid mechanics fields. A free tetrahedral mesh, automatically generated by the system, is employed. The maximum element size is ensured to be smaller than one-fifth of the shortest acoustic wavelength. Surface integral operators are applied at Reference Planes I and II. A frequency-domain solver is selected for parametric analysis. This study focuses on low-frequency airborne noise control, with a frequency range of 100–1600 Hz and an appropriate frequency step size.

**Table 1:** Material properties used in the COMSOL simulation

Material	Density (g cm^−3^)	Young modulus (Pa)	Poisson ratio
EVA film	2.05	1.7 × 10^8^	0.45
HMW10	0.141	1.82 × 10^9^	0.1
HMW20	0.155	7.62 × 10^8^	0.1
HMW30	0.208	4.26 × 10^8^	0.1

#### Molecular Dynamics Simulation

Molecules were randomly inserted into a 4 nm × 4 nm × 6 nm simulation box in the corresponding numbers according to the molar ratio of cellulose: hemicellulose: lignin of 29:4:1, and then a water box with the size of 4 nm × 4 nm × 8 nm was filled at the bottom of the box, and then the simulation box was expanded to 4 nm × 4 nm × 16 nm to construct system 1. The procedure for system 2 is the same as above, System 2 contains 29:4:1:1 cellulose: hemicellulose: lignin: AESO molecules. Then the molecular dynamics simulation of the systems was carried out. All-atom MD simulations were conducted using the GROMACS software package, version 2021.5. The gromos54a7 force field was employed to describe the molecules. First, the system was subjected to energy minimization using the steepest descent method to address initial contact issues. Subsequently, a data production run lasting 50 ns was performed using the NPT ensemble. In this process, all macromolecules except water molecules were fixed. The process used a Berendsen constant pressure to maintain the pressure at P = 1.0 bar and the temperature was controlled at 298.15 K using a velocity-rescale thermostat with a coupling constant of τ = 0.1 ps. Nonbonded interactions were computed with a cutoff of 1.2 nm, and long-range electrostatic interactions were computed using the particle-mesh Ewald summation method. All hydrogen bonds were constrained using the LINCS algorithm. Simulations were performed with a time step of 1 fs, and the neighbor list was updated every 10 steps. Periodic boundary conditions were applied in all three directions.

## Results and Discussion

### Morphology and Chemical Properties of HMW

**Fig. 2:**
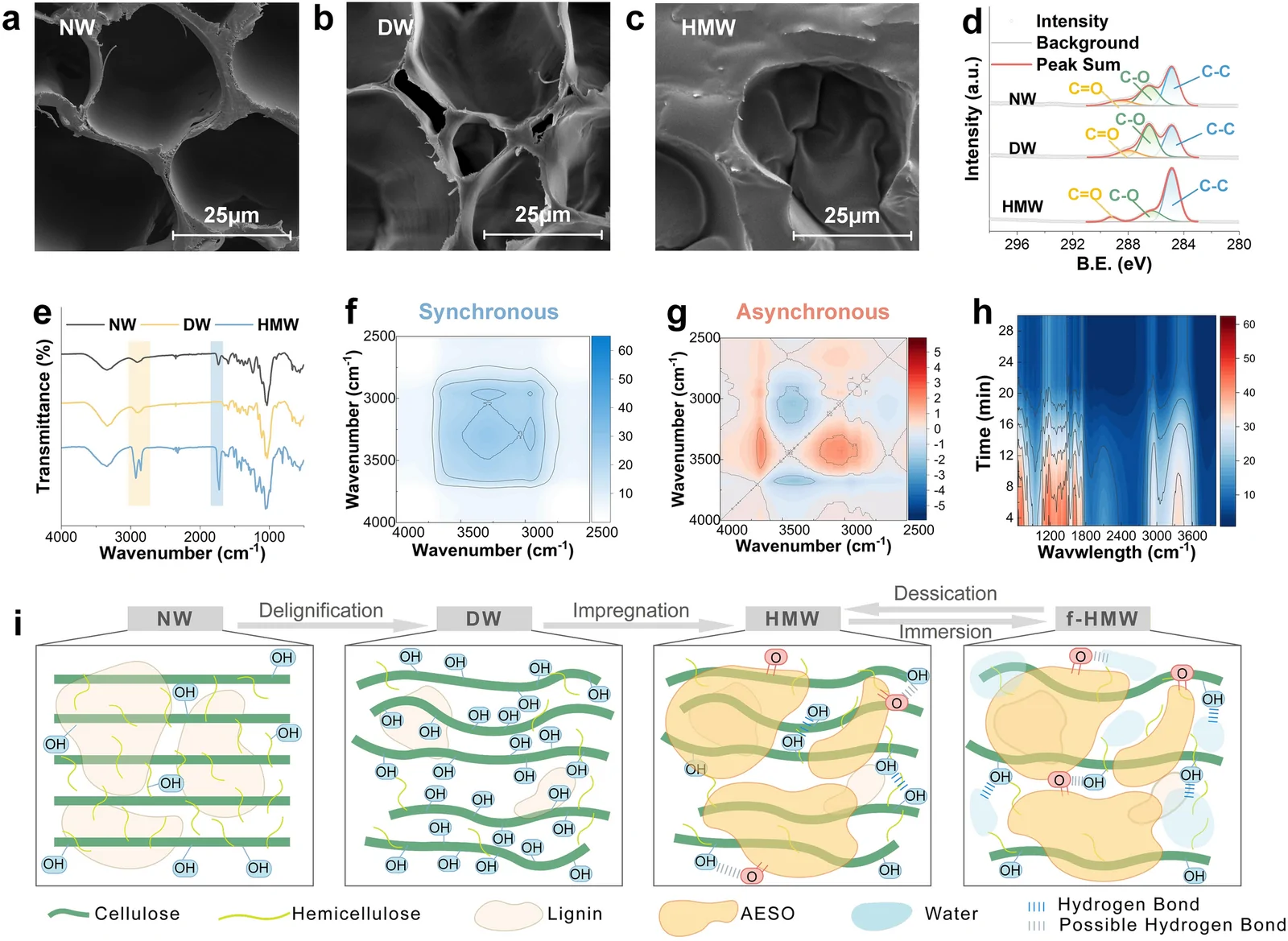
Microstructure and surface chemistry characterization of HMW. **a**-**c** SEM images of NW, DW, and HMW. After delignification, more pores appear in the DW, while the pores are filled after impregnation. **d** C 1*s* XPS spectra of NW, DW and HMW. **e** FTIR spectra of NW, DW and HMW. **f**-**g** Synchronous and asynchronous spectra of HMW. **h** 2D-IR spectra of HMW at 60 °C for 0–30 min. **i** Chemical preparation principles of delignification, impregnation, immersion and desiccation

The main chemical constituents of wood include cellulose, hemicellulose and lignin, each contributing distinctively to the properties of wood materials [23]. The natural basal wood exhibited a three-dimensional multi-layered porous cell structure, and the vessels are the main cellular units in cross-section [24]. Numerous studies have shown that the selective removal of lignin and hemicellulose enhances the porosity of wood [25–27]. To obtain a porous cellulose framework suitable for impregnation of AESO resin, we pretreated the material by partially removing lignin and hemicellulose. Subsequently, we impregnated the AESO resin into the wood through vacuum impregnation to ensure uniform filling degree of the wood pores, thereby obtaining highly moldable wood (HMW). HMW can realize 3D shaping by breaking and recombining hydrogen bonds during the wetting and drying cycles. Scanning electron microscope (SEM) images revealed the microscopic morphological features of natural wood (NW) (Fig. 2a), delignified wood (DW) (Fig. 2b), and HMW (Figs. 2c and S1). Lignin and hemicellulose were partially depolymerized to manufacture DW and the wood cell walls of DW shrank and became thinner, causing more microscopic pores to appear at the cell corners [24]. However, the macroscopic structure of *basal* wood remained intact, and its cellulose framework was preserved, thereby ensuring the structural integrity of the matrix material. The comparison chart of the contents of each component in NW and DW (Fig. S16) can verify the partial removal of lignin and hemicellulose. The contents of lignin and hemicellulose decreased by 4% and 7% respectively. In addition, the partial delignification process loosened the cellulose structure, increased the internal space of the material, and formed a more porous network structure, facilitating subsequent resin impregnation [28]. As evidenced by nitrogen sorption analysis, the specific surface area increased by 51% from 0.82 m^2^ g^−1^ for NW to 1.24 m^2^ g^−1^ for DW, driven by significant increases in micropore, mesopore, and macropore areas (Fig. S22).

Figure S1 showed the microscopic images of the composite material obtained by impregnating with different concentrations of AESO solution. As the concentration of AESO increased, more vessels were filled with resin. It should be noted that the cellular morphology of the wood did not deform after impregnation. The hollow wood vessels were partially filled with AESO when the concentration of AESO was low. Cell walls of fulfilled vessels were significantly thicker than those of DW. When the concentration of AESO increased to 30 wt%, the wood vessels were fully filled, indicating successful impregnation of AESO into the wood structure. Further increasing the impregnation concentration to 40 wt% did not result in any significant improvement in the filling condition of the wood vessels. The amount of residual resin after impregnation increased, resulting in certain waste. Therefore, a concentration of 30 wt% is selected as the optimal impregnation condition. The chemical compositions of NW, DW and HMW were analyzed using infrared spectroscopy technology. Figure 2e showed that a significant increased absorption peaks at 3340 cm^−1^ suggest enhanced exposure of hydroxyl groups, likely due to partial removal of hemicellulose and lignin. Notably, HMW displayed two characteristic peaks at approximately 2900 cm^−1^, the same as AESO [29], indicating the successful incorporation of the AESO compound into pretreated wood. Additionally, an absorption peak at 1730 cm^−1^ was observed in NW, representing the C = O bond mainly found in lignin [30]. The 1730 cm⁻^1^ peak in HMW corresponds to ester groups in AESO, distinct from lignin-related C = O bonds in NW. The peak in DW disappeared but was significantly enhanced in HMW, indicating the removal of lignin and the effective introduction of C = O bonds from AESO.

We performed in-situ infrared analysis on flexible HMW (f-HMW) which was the wet state of HMW. During the drying process at 60 °C for 30 min, variations in absorbance were observed to record synchronous and asynchronous infrared spectra (Fig. 2f, g). As shown in the 2D-IR plot (Fig. 2h), a decrease in absorbance at 3340 cm⁻^1^ was detected from 0 to 30 min, accompanied by a slight shift of the peak center toward lower wavenumbers. It was evident that as moisture was removed, the drying effect becomes more pronounced, leading to a progressive strengthening of the hydrogen-bond network within the system. This behavior may be attributed to the dehydration-induced structural reorganization of hydrogen bonding, which contributes to the shaping mechanism. Synchronous and asynchronous spectra (Fig. 2f, g) further demonstrate that the overall outcome of this dynamic process involves the reorganization of the hydrogen-bond network throughout the entire hydroxyl region. Under constant drying conditions, hydroxyl groups with weaker hydrogen-bonds underwent bond breakage initially, followed by subsequent changes involving hydroxyl groups with stronger hydrogen-bonds. To quantitatively substantiate the hydrogen-bond domain reconstruction, the O–H stretching region (3600–3200 cm⁻^1^) of the *in-situ* FTIR spectra was deconvoluted. The broad band was resolved into five sub-peaks corresponding to distinct hydrogen-bonding states: free/weak (~ 3555 cm⁻^1^), weak-medium inter-chain (~ 3490–3516 cm⁻^1^), medium-strong intra-chain (~ 3422–3443 cm⁻^1^), and strong cooperative bonds resembling cellulose α-phase (~ 3360–3362 cm⁻^1^) and β-phase (~ 3279–3289 cm⁻^1^) (Fig. S19). Quantitative analysis revealed a systematic shift during drying: the population of strong cooperative hydrogen bonds increased by 14.33%, while free OH groups decreased. This redistribution, coupled with a red-shift in the band center indicative of strengthened average hydrogen-bond energy, directly correlates with the macroscopic shape fixation, confirming that drying drives a preferential reconstruction towards a stronger, more stable hydrogen-bond network responsible for locking the 3D configuration.

The drying process at 60 °C represents a dynamic sequence of H-bond network disruption and reformation within the f-HMW. Initially, moisture reduction disrupted weaker water-mediated H-bonds (Fig. 2i). This dehydration process liberated polymer chains and induced a concurrent concentration effect. Driven by thermal energy at 60 °C, the enhanced chain dynamics facilitated a precise molecular rearrangement. Subsequently, within the increasingly concentrated system, the hydroxyl groups of cellulose reoriented. Crucially, this enabled the reformation and strengthening of the intrinsic cellulose-cellulose hydrogen-bond network. Concurrently, based on the quantitative spectral changes, hydrogen bonds between cellulose hydroxyls and AESO carbonyl groups are likely established. Deconvolution analysis revealed that the population of strong cooperative hydrogen bonds increased, while free OH groups decreased. This redistribution, coupled with a progressive red shift of 12–18 cm⁻^1^ in the hydroxyl stretching band center (Tables S1 and S2), quantitatively confirms a net enhancement in the average hydrogen-bond strength. The disruption of weaker, water-mediated bonds thus enabled the optimization of the overall hydrogen-bond network, comprising both the reconstituted cellulose matrix and the newly formed cellulose-AESO interface. This reconstructed and strengthened network, as illustrated in the refined Fig. 2i, constitutes the fundamental basis for the 3D shaping capability and its subsequent dimensional stability.

The X-ray photoelectron spectroscopy (XPS) test results (Fig. 2d) were consistent with this result. When lignin and hemicellulose were partially removed, more hydroxyl groups were exposed in basal wood, and the proportion of C-O bonds increased by 10% compared with NW, providing more binding sites for subsequent AESO impregnation. Compared with DW, the proportion of C-O bonds in HMW decreased by 23%, while the proportion of C–C bonds increased by 31.5%. The combined FTIR and XPS analyses indicate that AESO is successfully incorporated into the wood framework. In concert, the systematic redshift observed in both the C = O stretching vibration peak (~ 1730 cm⁻^1^) and the O–H stretching region in FTIR provides direct evidence for the formation of strong hydrogen-bond interactions Thus, we hypothesized the chemical preparation principles of HMW (Fig. 2i). This finding shows that partial removal of lignin and hemicellulose creates a porous network, facilitating deep resin infiltration while preserving structural framework of cellulose. As an amorphous substance, AESO undergoes chemical cross-linking with DW and physically dispersed between wood fibers. When water enters the HMW and damages the cross-linking networks between fibers and AESO resin, it temporarily causes the material to swell and gain plasticity before the connection is rebuilt by drying and water loss. This restructured interface, dominated by hydrogen bonding and physical intertwining, protects the cellulose framework and is fundamental to the shaping behavior and stability of HMW.

### Hydrogen-Bond Assisted Plasticity of HMW

**Fig. 3:**
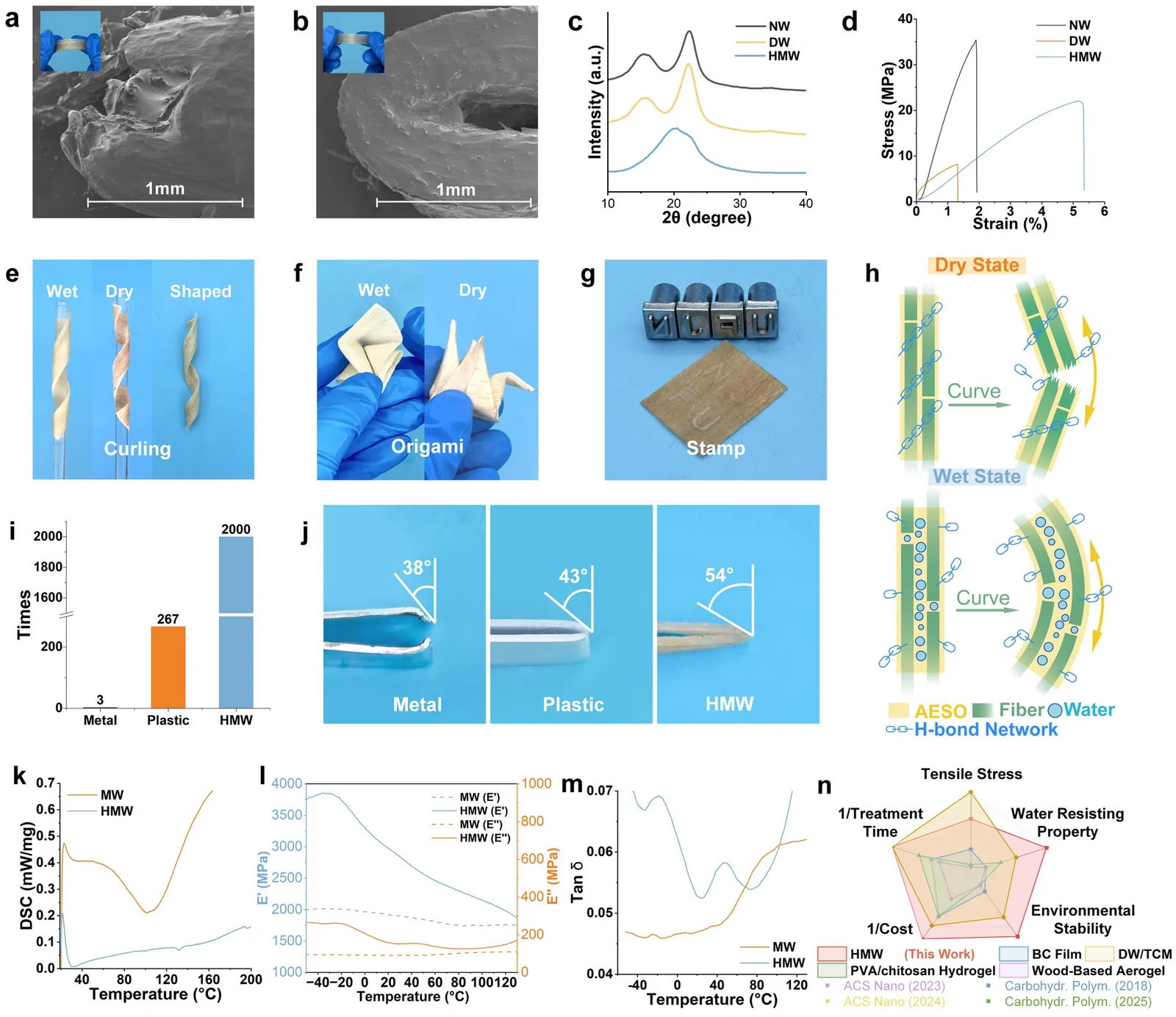
Plasticity property of HMW. **a**-**b** SEM images of HMW and f-HMW, images in the upper right corner are pictures demonstrating the creases of the two materials. **c** XRD spectra of HMW showing the increase of amorphous substances. **d** Stress–strain curve of NW, DW and HMW. **e** Winding process of HMW. **f** Origami process of HMW. **g** Molding pattern production process through water-asisted molding. **h** Principle of water-assisted shaping. **i** Foldable times of metals (aluminum plates), plastics (PET), and HMW. **j** Ultimate bending angle at which the material fractures during repeated folding tests.** k** DSC test curve graph. **l-m** DMA test result graph shows E', E" and tanδ, which represent the storage modulus, loss modulus and loss factor of the material respectively. **n** Property comparation of other flexible biomaterial [33–36]

To demonstrate the capabilities of HMW, we integrated traditional and advanced three-dimensional shaping techniques, including winding, origami, and molding, to systematically explore the exceptional 3D programmability of HMW. However, in the dry state, the plasticity of the HMW is still insufficient to support the intricate shaping operation with a relatively high degree of bending. When the dry HMW samples were folded, obvious fiber fractures occurred (Fig. 3a). At the same time, there was no fiber fracture in wet f-HMW (Fig. 3b) and HMW maintained an well-preserved fiber arrangement structure. Compared with NW and DW (Fig. S3), f-HMW exhibited excellent foldability and flexibility while establishing a robust foundation for its potential as a moldable material. Figure 3h explains the principle of hydrogen-bond assisted plasticity. In the dry state, the fibers are closely arranged, and the components form a relatively rigid structure through hydrogen-bond networks. However, in f-HMW, water molecules penetrate fiber gaps, breaking the hydrogen bonds between fibers and causing structural disintegration [12]. In such situation, adjacent fibers slid past each other without being torn. During the drying process, water molecules evaporated, and the hydrogen bonds between fibers and AESO were reestablished, resulting in a tighter structure and achieving the 3D shaping effect.

XRD was used to evaluate the changes in cellulose crystallinity (Fig. 3c). The CrI, calculated via the Segal method, was 61.72% for NW and 63.06% for DW. The slight increase for DW is attributed to the removal of amorphous lignin and hemicellulose [31]. In contrast, for HMW, the characteristic cellulose I peaks were largely obscured, leaving a broad peak at 20.36°. The extensive impregnation of AESO creates a dominant amorphous phase, making the distinct amorphous baseline at ~ 18° required for the Segal calculation indistinguishable. The impregnation of AESO disrupted the crystallinity of the wood structure, leading to the swelling of fibers and a decrease in mechanical properties but significantly improving plasticity. This observation was consistent with the results of mechanical property testing (Fig. 3d). Mechanical tensile tests confirmed the improved flexibility of the material, with HMW exhibiting greater strain before fracture. Moreover, higher strain-to-failure confirms enhanced plasticity. Under pressure and desiccation, the wood microscopic honeycomb structure [32] was compressed but maintained structural integrity through its cushioning effect. This stress-induced compression instead generated a layered architecture, thereby enhancing the mechanical properties of the material compared with DW (Fig. S2). As illustrated in Fig. 3e, HMW were wound onto a glass rod substrate and processed in an oven for drying and shaping, resulting in a stable helical configuration that retained its structural integrity for over 10 months from -5 to 40 °C without deformation. To evaluate the long-term shape stability under load, a basic creep resistance test was conducted. A strip of HMW was subjected to a constant tensile stress by suspending a 200 g mass at ambient conditions. Over a period of 1 week, no measurable elongation or creep deformation was detected (Fig. S21). These combined short- and long-term observations demonstrate that the reconstructed hydrogen-bond network, coupled with the AESO matrix, effectively suppresses time-dependent viscoelastic flow, endowing HMW with excellent dimensional stability under sustained load. Figure 3g further exemplified the versatility through four little letter molds (“NJFU”) with 1 mm in width and 1 mm in thickness, where embossed patterns were precisely imprinted onto HMW surfaces after drying. This embossing technique highlights the potential applications of HMW in customizable decorative materials, enabling the fabrication of patterned sheets via hot pressing. Notably, Fig. 3f showcases the compatibility of HMW with traditional Chinese origami art. A square wood sheet underwent sequential folding and drying to achieve an intricate “thousand-paper crane” structure, underscoring precision processability of HMW. To further verify the bending performance of the materials, we conducted repeated folding tests on metal (aluminum sheet), plastic (PET), and HMW (Fig. 3i). The test results showed that after 2000 folds of 180°, HMW still did not show obvious fractures (Fig. S4), while the aluminum sheet and PET (0.5 mm thickness) could only withstand 3 and 267 folds respectively. At the same time, by analyzing the ultimate bending angle of the materials after fracture (Fig. 3j), it can be found that the folding effect of HMW is superior to the other two materials, being able to withstand a bending angle of 54°. SEM of the permanent crease (Fig. S18) showed fiber reorientation but an absence of microcrack, directly demonstrating the ability to undergo severe plastic deformation via hydrogen-bond dynamics without structural fracture. The study on the tensile strength of the material after folding (Fig. S20) shows that the number of folds has the least impact on the tensile strength of HMW. After only 5 folding cycles, the tensile strength of the metal control (aluminum) decreased sharply by 47.5%. The plastic (PET) retained better strength but still suffered a 14.57% loss after 50 cycles. In stark contrast, HMW demonstrated exceptional fatigue resistance, with only a 6.75% reduction in tensile strength after 50 cycles. This quantitative comparison underscores that HMW maintains its mechanical integrity far more effectively under repeated deformation than conventional malleable materials.

To further compare with other moldable woods (MW), we conducted DSC and DMA tests. The DSC diagram (Fig. 3k) clearly compares the differences in hygroscopicity between the two modified woods. Due to its hydrophilic structure, MW exhibits a huge endothermic peak of water evaporation during heating. However, HMW impregnated with AESO is generally insensitive to humidity due to the hydrophobic barrier effect of the resin, and its heat flow curve is gentle. Meanwhile, the DMA test results show that the dynamic mechanical properties of wood impregnated with glue (HMW) have been comprehensively enhanced compared with lignin-free wood (MW). As shown in Fig. 3l, the energy storage mode (E') of HMW is significantly higher than the orange curve (MW) throughout the entire temperature range. This indicates that the impregnated colloid forms a strong support network between the fibers, greatly enhancing the overall stiffness of the composite material. The loss modulus (E") measured in Fig. 3l reveals the viscosity and internal friction characteristics of the material. The loss modulus value of HMW is comprehensively higher than that of MW. The HMW curve gradually rises after approximately 80 °C, due to the glass transition of the impregnated resin. The resin forms a continuous phase with strong molecular motion capabilities in the matrix, enabling HMW to convert more mechanical energy into thermal energy and dissipate it when subjected to external forces, thereby demonstrating superior shock absorption and impact resistance potential. Figure 3n characterizes the loss factor tanδ of the material. The loss factor value of HMW is consistently higher than that of MW throughout the process and presents a very prominent damping peak at approximately 90 °C. A higher tanδ value means that HMW has a more outstanding damping capacity, that is, better vibration absorption and noise reduction effects. This feature makes HMW more advantageous than MW in application scenarios that require shock absorption. As a result, HMW is a highly suitable moldable material for 3D structures, offering balanced mechanical strength, environmental stability, and flexibility compared to similar moldable woods.

Additionally, we have designed a large-scale and continuous production scheme. By rolling HMW into coils and then subjecting them to plastic deformation in a continuous press (Fig. 1e), this production method significantly enhanced the possibility of industrial application of HMW. Meanwhile, large-sized samples (500 mm in length) could also be fabricated (Fig. 1d), indicating that HMW holds great application potential. The results above confirmed the plasticity of HMW and its potential to replace moldable or meltable materials, such as metal and plastic. When applying flexible biomaterials to the sound insulation layers of long-distance transportation equipment such as high-speed trains, the material must achieve a stringent balance between mechanical properties, water resistance, and environmental stability. In such application scenarios, there is a risk of reduced sound insulation performance due to moisture absorption-induced expansion or structural failure. As shown in Fig. 3n, the HMW developed in this study demonstrates significant advantages compared with other flexible biomaterial [33–36]. While maintaining excellent mechanical properties, its superior hydrophobic effect and exceptional environmental stability ensure long-term dimensional stability of the sound insulation structure under harsh conditions, preventing performance degradation. Meanwhile, HMW has the potential for industrial production due to its simple process and low cost. This establishes a foundation for its use as a reliable and durable structural material in highly demanding industrial applications such as high-speed trains.

### Environmental Stability of HMW

Wood possesses the inherent property of swelling while absorbing water and shrinking while desorbing water. Due to its inherent non-uniformity, it is prone to cracking under the influence of the environment, which affects the environmental stability of wood materials. Nevertheless, materials used for advanced 3D structures need to cope with more severe environmental challenges, such as temperature and humidity changes during long-distance transportation or low-temperature and low-pressure environments at high altitudes, requiring engineering materials to have excellent environmental stability. In this study, we impregnated AESO to reconstruct the hydrogen bonds which connect the cellulose fibers and reduce the exposed hydroxyl groups. This approach can protect the cellulose skeleton and reduce the impact of environmental changes on the wood structure.

**Fig. 4:**
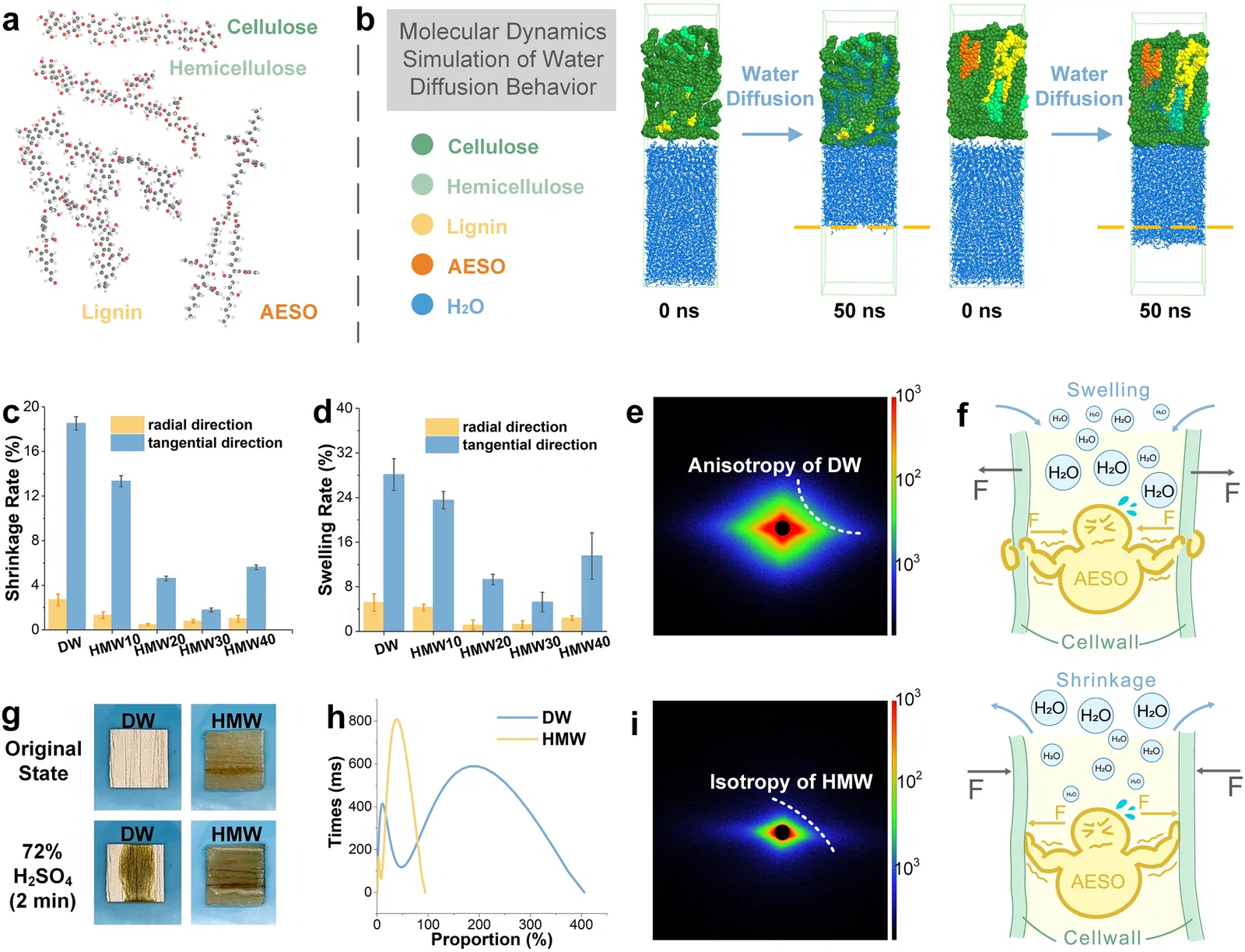
Dimensional stability of HMW. **a** Molecular model of cellulose, hemicellulose, lignin and AESO. **b** Molecular dynamics simulation of water diffusion behavior. The model on the left shows DW and the one on the right shows HMW. **c** Shrinkage rate of DW and HMW. **d** Swelling rate of DW and HMW. **e** and **i** SAXS images of DW and HMW. **f** Principle of high dimensional stability. **g** Drop concentrated sulfuric acid on the material surface to demonstrate the acid resistance of DW and HMW. **h** Low-field nuclear magnetic resonance (L-NMR) curves of DW and HMW

In order to explore the mechanism of improving the dimensional stability of HMW in this study, the following systems were constructed by means of molecular dynamics simulation. The simulation probed water diffusion in HMW (Fig. 4b). Figure 4a shows the molecular structure of each component in the molecular dynamics simulation. After 50 ns, water molecules hydrated cellulose fibrils, inducing temporary hydrogen bond breakage for enhanced plasticity. The low dimensional stability of wood is attributed to the free hydroxyl groups in the amorphous region of cellulose, forming hydrogen bonds with water molecules in the air. The AESO resin occupied the transport path of water molecules and caused fewer water molecules to enter the system, and the water molecular diffusion coefficient was 4% lower than that of DW. This system minimized moisture-induced dimensional changes while enhancing plasticity as the amorphous material AESO replaced water to build new hydrogen bonds. The results of low-field nuclear magnetism also indicated that the relative content of bound water in HMW was lower than that in DW [37], which meant that the influence of water on dimensional stability is greatly weakened (Fig. 4h).

Figure 4c and 4d illustrated the shrinkage rate and the swelling rate of different wood. The data of each sample was measured three times and the average value was taken.The shrinkage rate and swelling rate of HMW30 decreased 90.36% and 81.28% compared with DW, showcasing its greatly improved dimensional stability. As the impregnation concentration increased, the dimensional stability improved. When the impregnation concentration exceeded 30% and reached 40%, the fluidity of the AESO solution was too poor to penetrate deep into the wood, resulting in decreased dimensional stability of HMW40 due to the uneven impregnation. The analysis of the water contact angle (WCA) for NW, DW and HMW revealed that DW fully absorbed water within 1 s, with a WCA of zero. However, HMW remained unabsorbed even after 1 min, indicating that HMW exhibited dimensional stability (Figs. S5 and S17). The AESO, possessing certain hydrophobic properties, disrupted the hydrogen bonds within the material. This resulted in a reduction in hygroscopicity and an enhancement of its stability. Figure S6 shows the rapid water absorption effect of DW and the excellent stability of HMW when droplets of water are simultaneously applied to the surface of the wood chip. After 60 s, the water droplets on the surface of the DW chip were completely absorbed, while the water droplet morphology on the surface of the HMW chip remained intact. Meanwhile, we also conducted acid and alkali resistance tests. When 72% H_2_SO_4_ droplets fell on DW, after 2 min, the surface of the wood chips showed obvious oxidative discoloration, while HMW showed no obvious color change (Fig. 4g). The HMW was further immersed in 10% H_2_SO_4_ solution and 20% concentration NaOH solution for 24 h and there was no obvious change in the appearance of the sample (Fig. S7). From the above molecular dynamics simulations and water influence experiments, it could be seen that the successful impregnation of AESO in HMW had replaced the original hydrogen bond binding sites between cellulose fibers. Thereby achieving a certain hydrophobic effect and extremely strong stability in different environments.

The SAXS images (Fig. 4e, i) revealed elliptical scattering patterns, indicating the anisotropy of DW. This anisotropy indicates that wood has a certain degree of directionality in its structure related to the orientation of fibers. No significant change was observed in the scattering orientation before and after impregnation, indicating that the epoxy acrylate impregnation had no significant effect on the overall structural orientation of the wood. However, the wood after impregnation (Fig. 4i) showed more concentrated and stronger scattering signals in terms of scattering intensity and distribution. This phenomenon might be attributed to the formation of a uniform network structure from AESO impregnation in the pores and cell walls of the wood. The uniform network increased the density and filling of the pores in the wood, improving the isotropy of the material [38]. Notably, this improved isotropy manifests in small-angle X-ray scattering (SAXS) as a more diffuse and circular symmetric pattern, contrasting with the anisotropic scattering typically seen in DW. As a consequence, the enhanced structural uniformity and reduced directional variations improved internal dimensional stability of the HMW. Quantitative analysis of the SAXS data provides definitive metrics for the nanostructural evolution (Table S1). The specific surface area (S/V) of HMW decreased by ~ 35% compared to DW, confirming the effective filling of nano-pores by AESO. More profoundly, the scattering invariant (Q) decreased by ~ 76%, indicating a drastic reduction in electron density contrast between the wood matrix and the filler. This signifies that AESO does not merely occupy voids but forms an intimately integrated composite phase with cellulose. Concurrently, a slight increase in the radius of gyration (R_g_) suggests a minor expansion of the dominant scattering entities, likely due to polymer coating or the formation of new interfacial regions. These quantitative parameters collectively evidence a transition from a discrete, porous, and anisotropic architecture (DW) to a continuous, dense, and more isotropic nanocomposite (HMW), which is the nanoscale foundation for the drastically improved dimensional stability.

The principle of good dimensional stability in the material is illustrated in Fig. 4f. When HMW absorbed water, it expanded. However, the presence of AESO weakened the swelling action of the wood cell wall by reducing the hydrogen bonds among cellulose fibers. During the drying process of the sample, water exited the vessels and the AESO resin formed tight bonds among cellulose fibers, hindering contraction in the wood. As a result, this hydrogen-bond substitution behavior of AESO allows HMW to exhibit high dimensional stability compared with other moldable wood. The above analysis confirmed the effectiveness of using AESO for impregnation to improve the dimensional stability of HMW. The demonstrated greatly improved dimensional stability of HMW positions it as a transformative candidate for advanced engineering systems, particularly in extreme transportation scenarios requiring greatly improved dimensional stability and acoustic performance. Therefore, HMW is more suitable for the sound insulation wall frame of aerospace aircraft or bullet trains and through its deformation, HMW can also better adapt to complex engineering structures.

### Metamaterial Structures Made of HMW

**Fig. 5:**
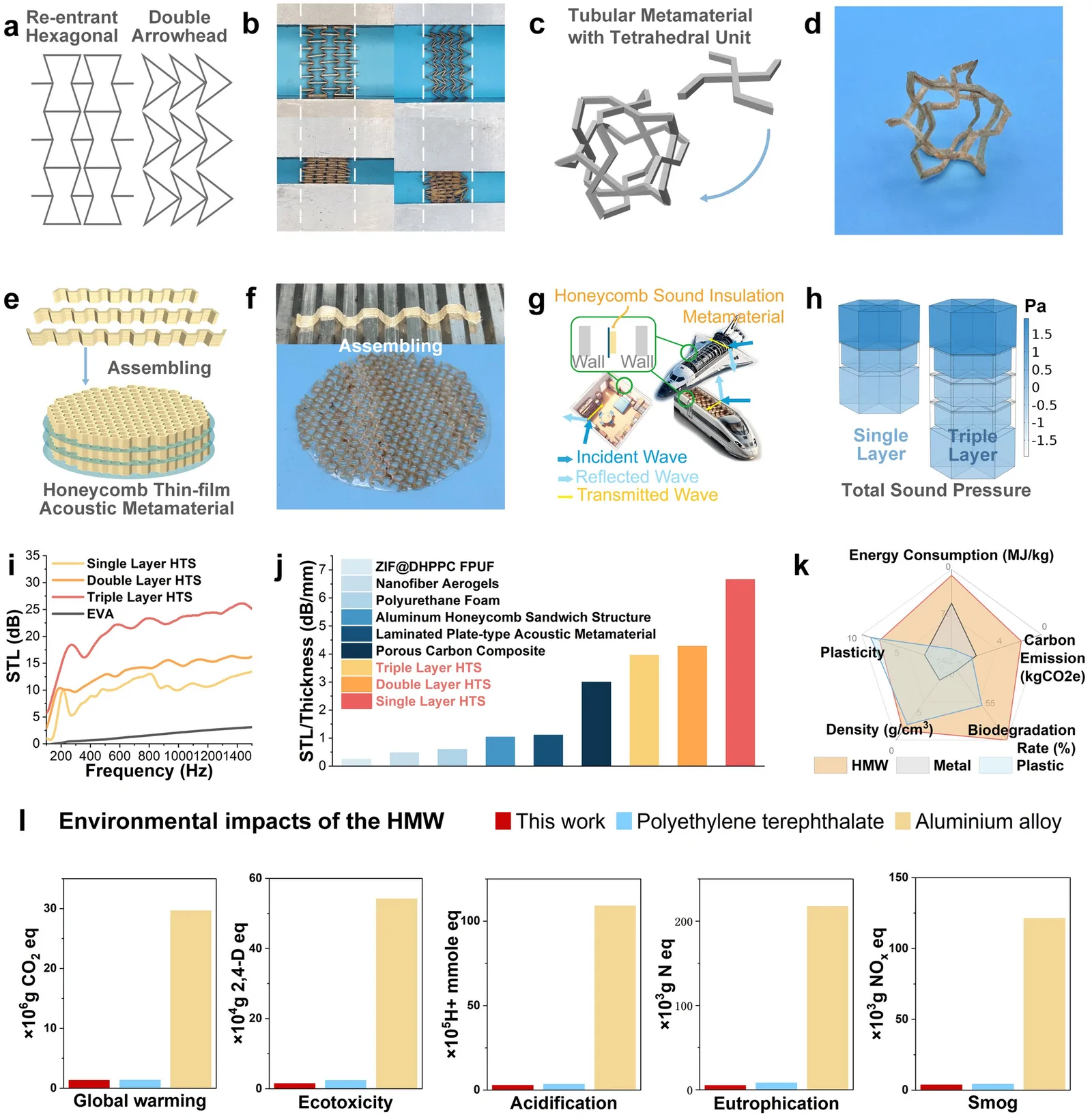
Different metamaterials made by HMWs. **a** Metamaterial of re-entrant hexagonal honeycomb structure and double arrowhead honeycomb structure. **b** Compression process of re-entrant hexagonal honeycomb structure and double arrowhead honeycomb structure. **c-d** Tubular metamaterial with tetra chiral unit. **e**–**f** Assembling process of HTS. **g** Application diagram of the sound insulation honeycomb structure. HMW can be applied to high-speed railways, spacecraft and house walls. **h** Simulation of total sound pressure under the frequency of 1600 Hz. **i** STL experimental results for different numbers of HTS layers. **j** Sound insulation performance and thickness comparison between HMW and other sound insulation materials. **k** Comparison of HMW for industrial manufacturing with metals and plastics. **l** Comparison of environmental impacts with PET and aluminum alloy

We prepared two different honeycomb structures and one tubular metamaterial with tetrahedral unit to demonstrate the potential of HMW to manufacture metamaterials (Fig. 5a-d). Re-entrant hexagonal honeycomb structure and double arrowhead honeycomb structure are two classic metamaterial structures with negative Poisson's ratio. When these two honeycomb structures were compressed, they exhibited the opposite deformation behavior to conventional materials, contracting rather than expanding during compression (Fig. 5b and Movie S2) [38]. Meanwhile, we also made a classic chiral metamaterial with tetrachiral unit, which is constructed in three dimensions by six windmill-like planar structures (Fig. 5d). These results collectively demonstrate programmable shaping behavior and its viability for metamaterial structure.

This production technology can be applied to the production of practical honeycomb structures. A honeycomb structure was assembled with EVA film (Fig. 5e, f) to manufacture a honeycomb thin-film structure and evaluated for sound transmission loss using a four-sensor methodology. The low-frequency sound insulation performance of the sample was assessed. The results showed that the honeycomb thin-film structure exhibited a significant sound insulation peak in the low-frequency range. Compared with the single-layer EVA film (Fig. S8), the honeycomb thin-film structure demonstrated enhanced sound insulation capabilities. Furthermore, we observed that by stacking and combining honeycomb structures with varying impregnation concentrations, the low-frequency sound insulation effect improved as the number of stacked structures increased, resulting in a broader bandwidth. Notably, the triple-layered HMW attained an insulation value of 18 dB at 200 Hz within the low-frequency spectrum (Fig. 5i). Honeycomb thin-film structures produced sound-insulation effects under the excitation of sound waves due to the coupled vibrational behavior of the thin film-mass element system [39]. A 6.3 mm triple-layer HMW can achieve comparable sound insulation performance to a 30–35 mm thick polyurethane foam [40] and a 22 mm epoxy resin-filled aluminum honeycomb sandwich structure [41]. Compared with other materials, it has better sound insulation performance [42] (Fig. 5j). Additionally, it is approximately 80% lighter than steel plates with similar sound insulation properties.

We used COMSOL software in the structural mechanics module and acoustic module of single cell structure to establish the finite element simulation model, and simulated the multilayer structure of sound insulation effect, the simulation curve with the actual measured curve trend is consistent (Fig. S9). The fluctuation of the actual measured data is presumed to be the influence of the overall vibration after the installation of the sample. By simulating the total sound pressure at 1600 Hz, it is evident that both the single layer and triple layer HTS exhibit significant sound insulation effects. The structure of the film and honeycomb forms a mass-spring system. When the film vibrates, it interacts with the honeycomb structure, and the sound energy is converted into mechanical energy and dissipated to realize sound insulation.

Notably, the 1600 Hz sound pressure distribution in Fig. 5h shows that sound waves are significantly attenuated by multiple reflections and scattering of the cellular pore walls when passing through each layer, and each layer provides additional energy dissipation paths, resulting in layer-by-layer reduction of transmitted sound pressure. The sound wave was affected by the multilayer structure and dissipates step by step between layers resulting in higher sound insulation. The geometric properties of the honeycomb transformed the sound energy into structural vibration, which was dissipated into heat energy through the internal friction of the material. Additionally, the stress distribution in Supplementary Fig. 10 shows wide and uniform areas of high stress, indicating that the structure efficiently dissipates energy via bending deformation and inhibits sound transmission. Furthermore, we envisaged the application scenarios of this material (Fig. 5g). The structure is inspired by honeycomb membrane-type acoustic metamaterials (AMs) [43–46], resulting in a high sound insulation value with thickness of only 6.3 mm. This exploration not only advanced the application scenarios of moldable wood in high-precision processes but also offered novel insights and strategic directions for the structured utilization of wood. Meanwhile, the plasticity via reversible hydrogen bonding allows precise molding of resonant cavities (e.g., hexagonal units) for targeted low-frequency attenuation. The moisture-resistant AESO matrix minimizes hygroscopic noise interference, while its amorphous nature enhances vibration damping, converting acoustic energy into heat through internal friction (Fig. 5h). The graded impregnation (HMW10—HMW30) further enables multilayer designs for broadband insulation by stacking varied pore structures.

In conclusion, this research demonstrated the application potential of HMW through various 3D programming methods, which could be widely applied in both traditional industries and advanced engineering fields. HMW-based structures hold promise for practical engineering applications, such as interior acoustic panels in aerospace cabins, noise-reduction modules in high speed train compartments, and lightweight architectural sound-absorbing wall. The combination of low density, high acoustic performance, and environmental stability makes them suitable for demanding transportation and infrastructure environments. HMW offers significant potential for industrial utilization with low energy consumption and notable environmental benefits. While metal melting requires hundreds to thousands of degrees Celsius, plastic melting needs about 100–200 °C, and the formation of moldable HMW only requires oven drying at 60 °C or even at room temperature (Fig. 5k). The application practices have demonstrated the significant potential of HMW in advancing green industries and delivering environmental benefits. To further demonstrate the environmental advantages of HMW in industrial production, we conducted a life cycle assessment (LCA) analysis. As can be clearly seen from the LCA environmental impact diagram (Fig. 5l), HMW shows significant advantages in many dimensions of environmental impact compared with PET and aluminum alloy. The environmental impact values of HMW are at extremely low levels in many assessment indicators including global warming, acidification, ecotoxicity, haze, and eutrophication. Therefore, compared with traditional material, HMW has a significantly lower environmental burden throughout its life cycle and is a more environmentally friendly material choice.

## Conclusions

This study established highly moldable wood (HMW), fabricated via a simple two-step process, as a sustainable platform for advanced engineering structures. The low-energy consumption preparation method derived from the hydrogen-bond remodeling mechanism makes it possible to prepare 3D structures on a large scale in an environmentally friendly way. Exhibiting over 80% reduction in swelling/shrinkage rate versus conventional wood, the high moldability of HMW enables the direct manufacturing of complex geometries like mechanical metamaterials (MMAs). The unique combination of high formability, tunable performance, and sustainability positions HMW as a transformative alternative to metals and plastics in demanding structural applications, leveraging nature-inspired design and scalable processing to accelerate the shift toward carbon–neutral advanced manufacturing. This work not only demonstrates a sustainable material platform for advanced engineering but also opens avenues for applications in aerospace, transportation, and green construction. The industrial potential of HMW is underscored by its renewable material base and uniquely low-energy shaping process. While challenges such as uniform impregnation in thick sections and the design of continuous production lines remain, the successful fabrication of large panels demonstrates scalable feasibility. Addressing these challenges through process optimization will be the focus of future translational work.

## Supplementary Information

Below is the link to the electronic supplementary material.

Supplementary file1 (DOCX 6180 KB)

Supplementary file2 (MP4 3999 KB)

Supplementary file3 (MP4 1975 KB)
