# Supplementary material for: Eco-Friendly, Multi-Mode Processable Highly Moldable Wood Enabled by the Reconstruction of Hydrogen-Bonding Domain
Source: Nanomicro Lett. 2026 Mar 4;18:269. doi: 10.1007/s40820-026-02121-y (PMC12961088; doi:10.1007/s40820-026-02121-y)
Supplement: Supplementary file 1 — Supplementary file1 (DOCX 6180 KB) [file 40820_2026_2121_MOESM1_ESM.docx]

Supporting Information for

**Eco-Friendly, Multi-Mode Processable Highly Moldable Wood Enabled by the Reconstruction of Hydrogen-Bonding Domain**

Rui Yang^1,^ *, Linghui Qi^1^, Xiaoli Wu^2^, Zhipeng Liu^1^, Huiyang Bian^3^, Changlei Xia^1^, Changtong Mei^1^, Shuaicheng Jiang^4,^ *, Meng Yao^5,^ *, Jianzhang Li^4,^ *

^1^ Jiangsu Co-Innovation Center of Efficient Processing and Utilization of Forest Resources, International Innovation Center for Forest Chemicals and Materials, College of Materials Science and Engineering, Nanjing Forestry University, Nanjing 210037, P. R. China

^2^ School of Mechanical and Electronic Engineering, Nanjing Forestry University, Nanjing 210037, P. R. China

^3^ Jiangsu Provincial Key Lab of Sustainable Pulp and Paper Technology and Biomass Materials, Nanjing Forestry University, Nanjing 210037, P. R. China

^4^ State Key Laboratory of Efficient Production of Forest Resources & MOE Key Laboratory of Wood Material Science and Application, Beijing Forestry University, Beijing 100091, P. R. China

^5^ College of Materials Science and Engineering, Sichuan University, Chengdu 610065, P. R. China

*Corresponding authors. E-mail: [yangrui@njfu.edu.cn](mailto:yangrui@njfu.edu.cn) (Rui Yang); [jiangsc@bjfu.edu.cn](mailto:jiangsc@bjfu.edu.cn) (Shuaicheng Jiang); [yaomeng@scu.edu.cn](mailto:yaomeng@scu.edu.cn) (Meng Yao); [lijzh@bjfu.edu.cn](mailto:lijzh@bjfu.edu.cn) (Jianzhang Li)

**Supplementary Figures and Tables**

^
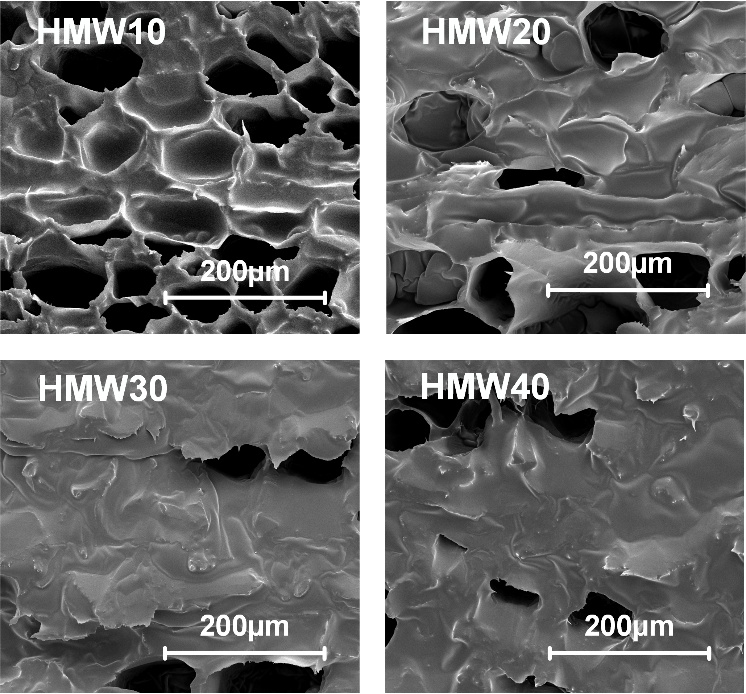
^

**Fig. 1** The SEM picture of HMW10, HMW20, HMW30 and HMW40


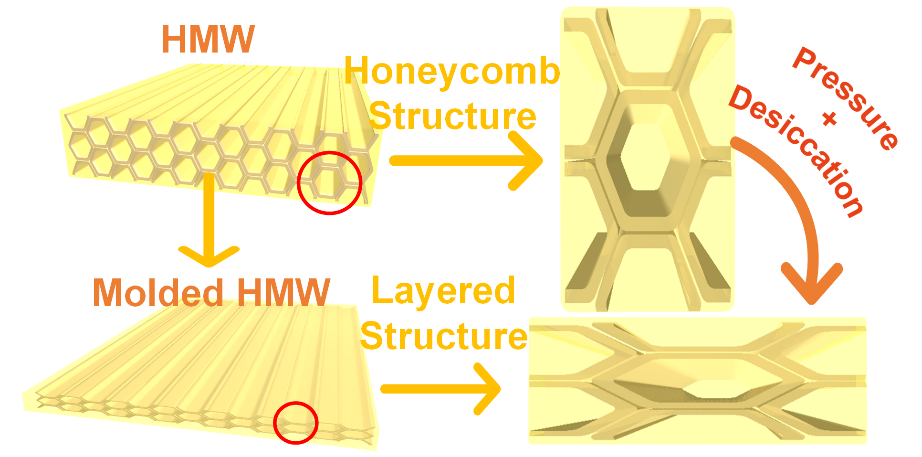


**Fig. 2** The compression principle of HMW


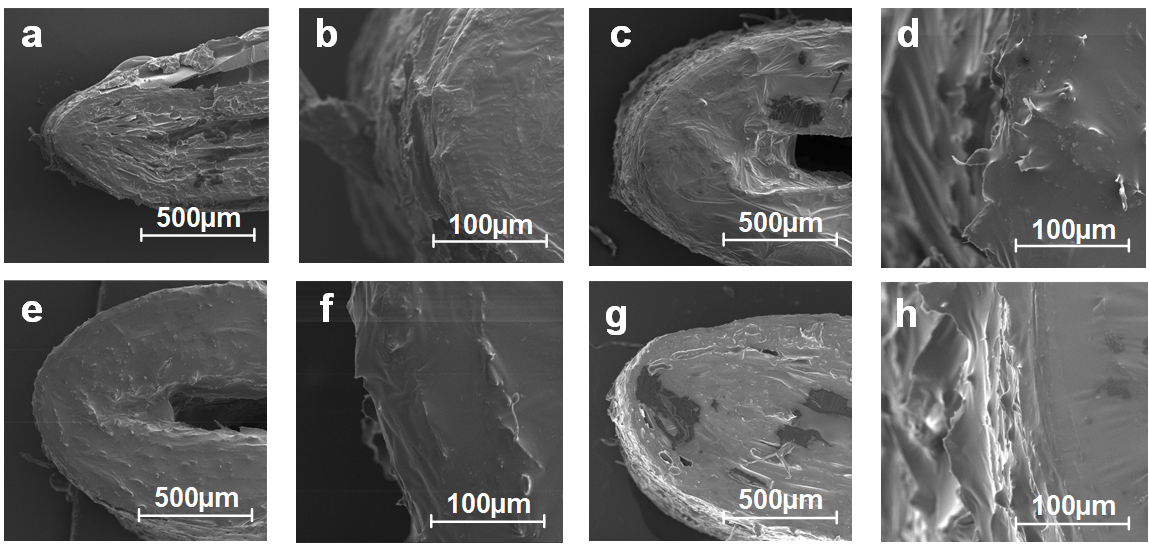


**Fig. 3** **a - b**, SEM image of HMW10. **c - d**, SEM image of HMW20;^
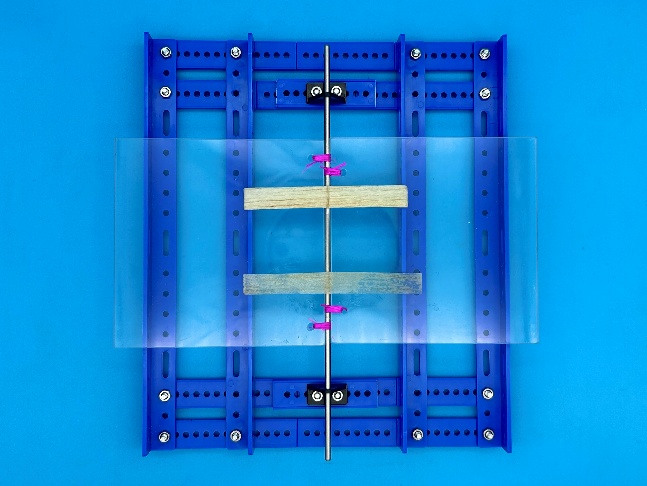
^ **e - f**, SEM image of HMW30; **g - h**, SEM image of HMW40


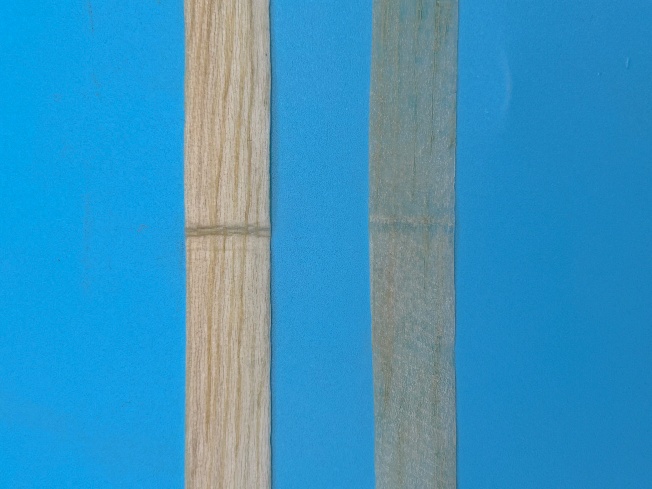


**DW**

**HMW**

**Fig. 4** Repeated folding test equipment and photos of samples after 2000 folds


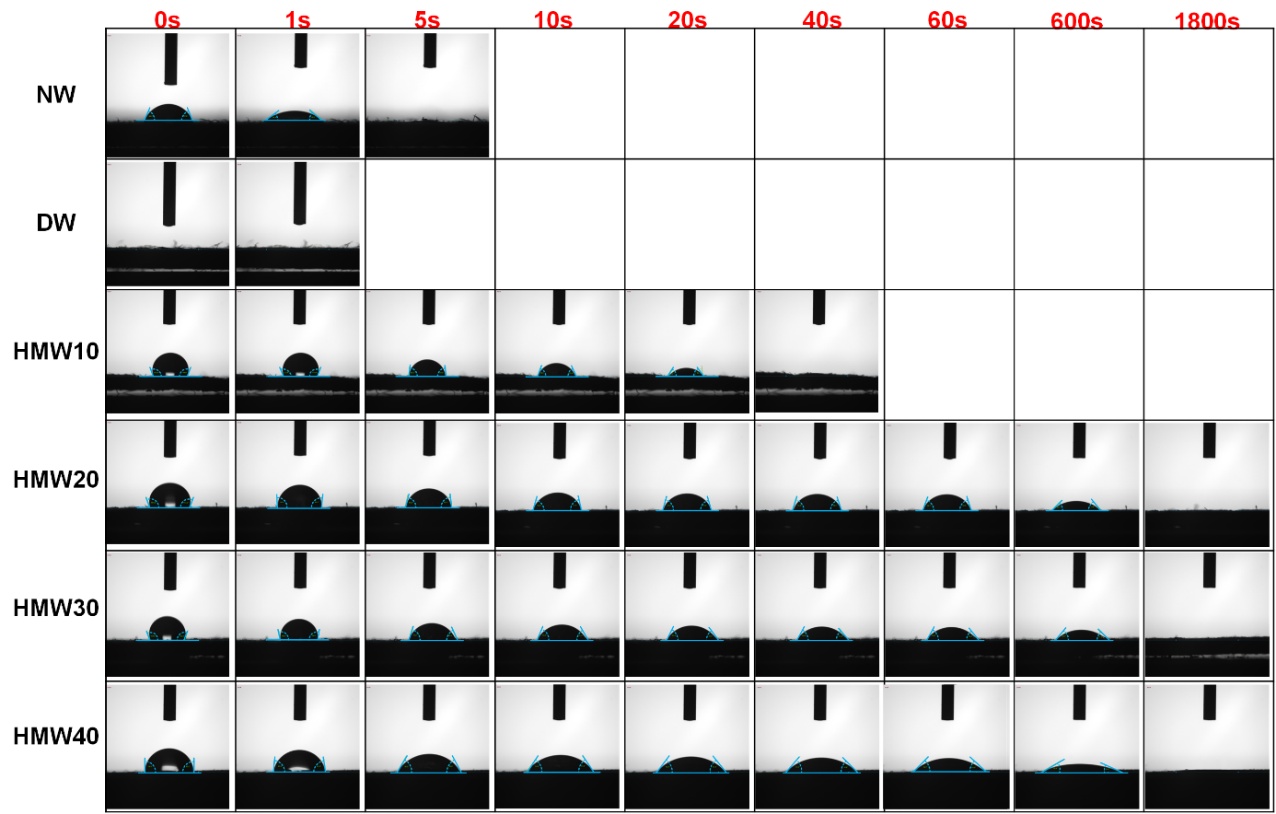


**Fig. 5** Water contact angle of NW, MW, HMW10, HMW20, HMW30, HMW40


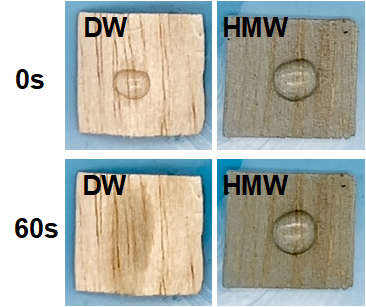


**Fig. 6** Water absorption effect of DW and HMW


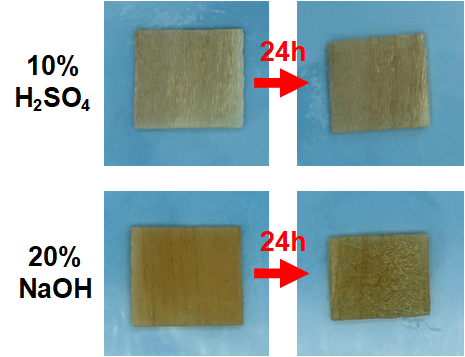


**Fig. 7** The acid and alkali resistance test of HMW

**Fig. 8** STL of honeycomb thin-film acoustic structures and EVA

**Fig. 9** The simulation curve and the actual measured curve of sound insulation test


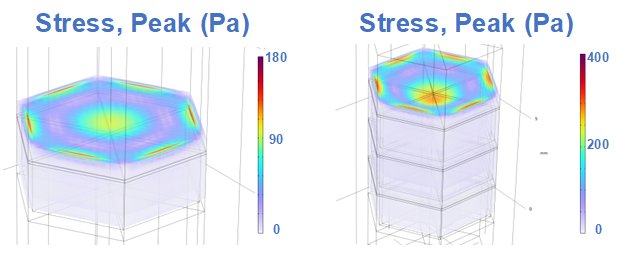


**Fig. 10** Vibration pattern of single layer structure and triple layer structure

**Fig. 11** The mass increasing rate of HMW10, HMW20, HMW30 and HMW40


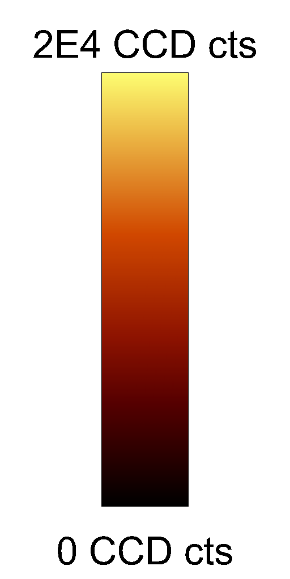

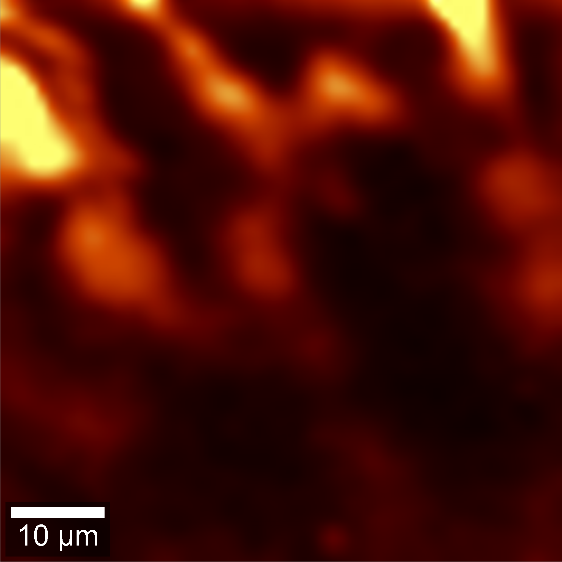


**Fig. 12** Raman imaging of HMW

**Fig. 13** Schematic diagram of impedance tube

DW

**Fig. 14** The MSD simulation curve of DW and HMW


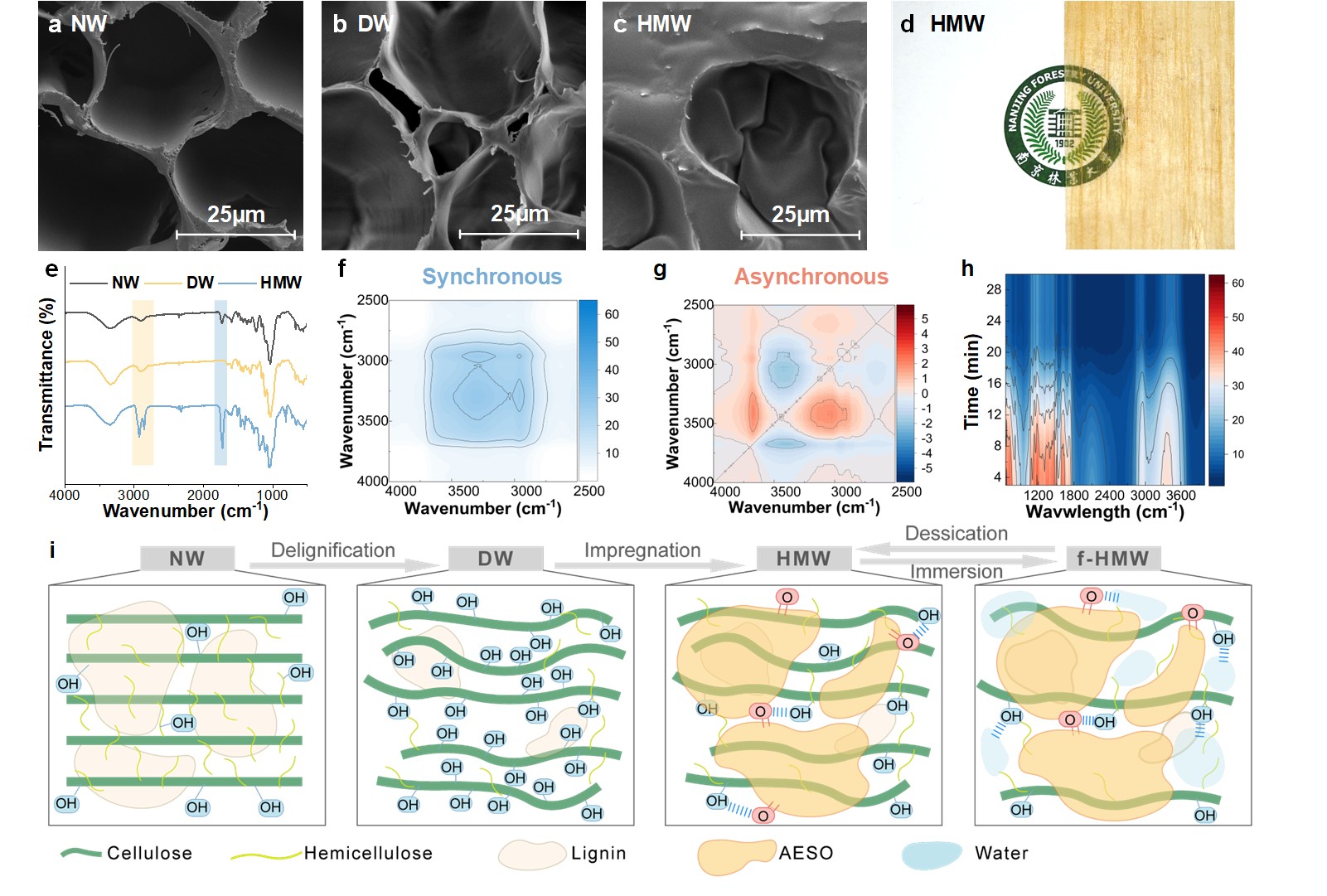


**Fig. 15** The semi-transparent effect of HMW

**Fig. 16** The composition content of NW and DW


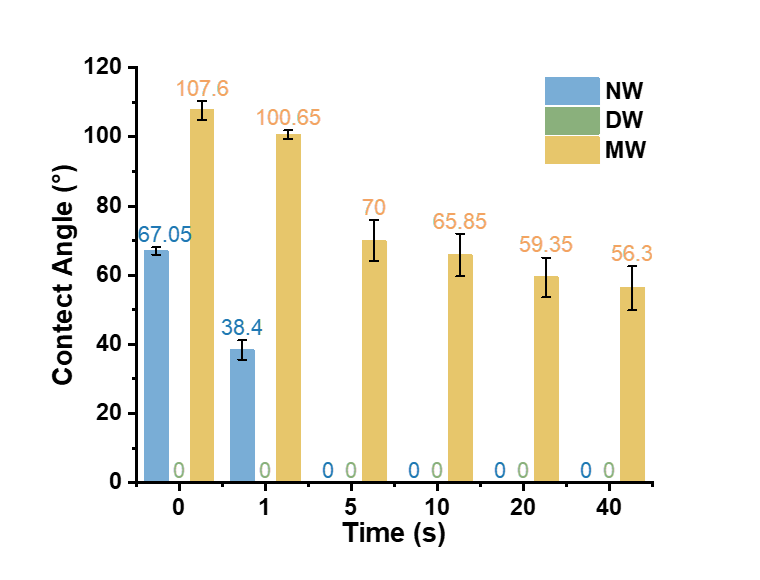


**Fig. 17** Water contact angles of NW, DW and HMW


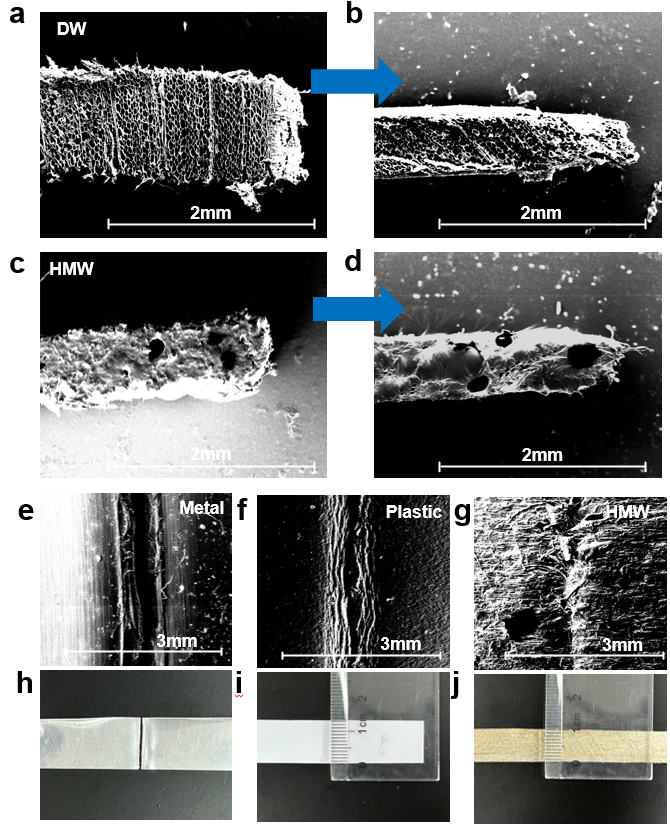


**Fig. 18** SEM images of the changes in HMW compression molding. **a** and **b** The pore changes of AESO without filling. **c** and **d** The pore changes during the shaping of HMW. **e** - **g** SEM images of creases after 50 folds for metal, plastic and HMW. **h** - **j** Photographs of creases after 50 folds for metal, plastic and HMW


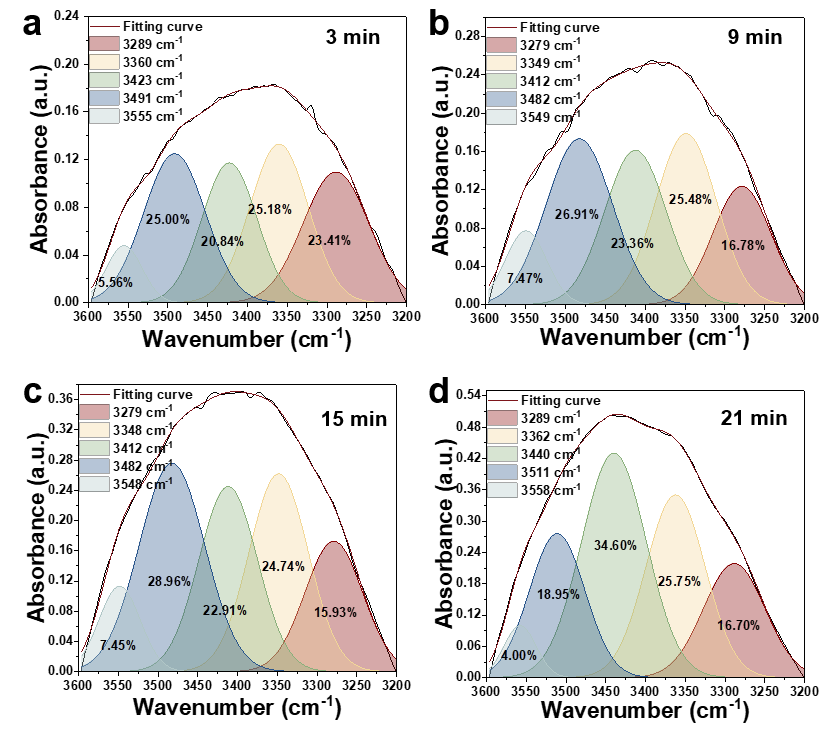


**Fig. 19** Peak deconvolution and fitting of HMW. **a** - **d** The hydrogen bond data of f-HMW after drying at 60 °C for 3, 9, 15 and 21 minutes were respectively presented

**Fig. 20** The influence of folding times on the tensile strength of materials


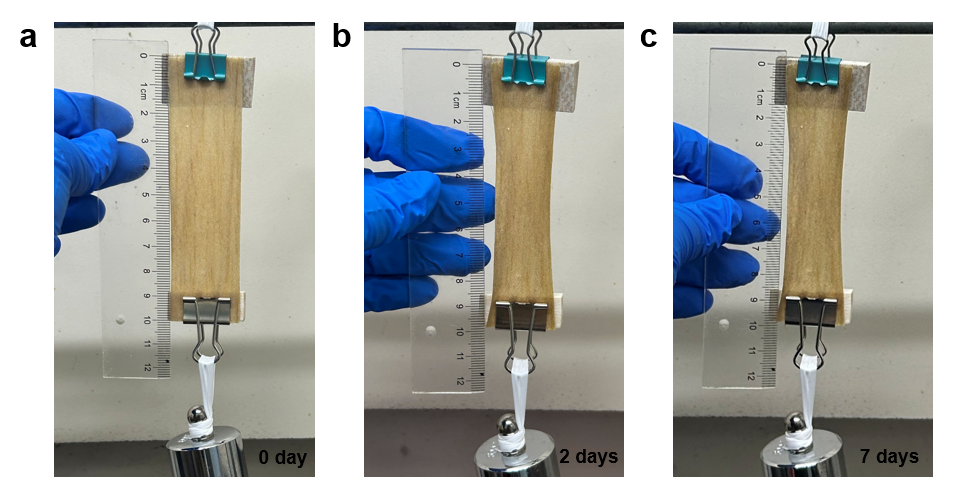


**Fig. 21** The tensile condition of HMW under long-term loading

**Fig. 22** The pore area distribution of NW and DW

**Movie S1** The plastic ability comparison of conventional DW (above) and HMW (below).

**Movie S2** The compression process of re-entrant hexagonal honeycomb structure.

**Table S1** Bond Energies and Bond Lengths of OW Samples Before and After Treatment

|  | [O(6)H…O(3’)] | | | [O(3)H…O(5)] | | | [O(2)H…O(6)] | | | O…H(2) | | | O…H(6) | | |
| --- | --- | --- | --- | --- | --- | --- | --- | --- | --- | --- | --- | --- | --- | --- | --- |
|  | BP | E_H_(kJ/mol) | R (Å) | BP | E_H_(kJ/mol) | R (Å) | BP | E_H_(kJ/mol) | R (Å) | BP | E_H_(kJ/mol) | R (Å) | BP | E_H_(kJ/mol) | R (Å) |
| 3 Min | 3288.74 | 25.98 | 2.77 | 3360.21 | 20.84 | 2.79 | 3422.62 | 16.35 | 2.80 | 3491.50 | 11.40 | 2.82 | 3554.83 | 6.84 | 2.83 |
| 9 Min | 3278.75 | 26.70 | 2.77 | 3348.68 | 21.67 | 2.78 | 3412.11 | 17.11 | 2.80 | 3482.45 | 12.05 | 2.81 | 3549.41 | 7.23 | 2.83 |
| 15 Min | 3279.19 | 26.67 | 2.77 | 3348.40 | 21.69 | 2.78 | 3411.81 | 17.13 | 2.80 | 3482.46 | 12.05 | 2.81 | 3548.22 | 7.32 | 2.83 |
| 21 Min | 3288.64 | 25.99 | 2.77 | 3362.34 | 20.69 | 2.79 | 3439.76 | 15.12 | 2.80 | 3511.06 | 9.99 | 2.82 | 3557.70 | 6.64 | 2.83 |

**Table S2** Deconvolution Results of the 3600-3200 cm^-1^ Band of OW Samples Before and After Treatment

|  | [O(6)H…O(3’)] | | [O(3)H…O(5)] | | [O(2)H…O(6)] | | O…H(2) | | O…H(6) | | R^2^ |
| --- | --- | --- | --- | --- | --- | --- | --- | --- | --- | --- | --- |
|  | FPAC | AR | FPAC | AR | FPAC | AR | FPAC | AR | FPAC | AR |  |
| 3 Min | 3288.74 | 23.41 | 3360.21 | 25.18 | 3422.62 | 20.84 | 3491.50 | 25.00 | 3554.83 | 5.56 | >0.99 |
| 9 Min | 3278.75 | 16.78 | 3348.68 | 25.48 | 3412.11 | 23.36 | 3482.45 | 26.91 | 3549.41 | 7.47 | >0.99 |
| 15 Min | 3279.19 | 15.93 | 3348.40 | 24.74 | 3411.81 | 22.91 | 3482.46 | 28.96 | 3548.22 | 7.45 | >0.99 |
| 21 Min | 3288.64 | 16.70 | 3362.34 | 25.75 | 3439.76 | 34.60 | 3511.06 | 18.95 | 3557.70 | 4.00 | >0.99 |
